# The effects of hand splinting in patients with early-stage thumb carpometacarpal joint osteoarthritis: a randomized, controlled study

**DOI:** 10.3906/sag-1807-157

**Published:** 2020-12-17

**Authors:** Aslı GENÇAY CAN, Nihal TEZEL

**Affiliations:** 1 Department of Physical Medicine and Rehabilitation, University of Health Science,Dışkapı Yıldırım Beyazıt Research and Training Hospital, Ankara Turkey

**Keywords:** Thumb osteoarthritis, splint, hand function

## Abstract

**Background/aim:**

Evidence for the effectiveness of splinting in thumb carpometacarpal osteoarthritis is limited. We aimed to evaluate the effects of a prefabricated carpometacarpal metacarpophalangeal immobilization splint on pain, hand function, and hand strength in patients with early-stage thumb carpometacarpal osteoarthritis.

**Materials and methods:**

Sixty-three hands with stage 1 or 2 thumb carpometacarpal osteoarthritis were enrolled in the study. The nonsplint group received oral information about how to accommodate daily activities. The splint group was given a prefabricated carpometacarpal metacarpophalangeal immobilization splint for 6 weeks. Pain was evaluated using the Australian/Canadian Osteoarthritis Hand Index (AUSCAN). Hand functions were evaluated using the AUSCAN and the Quick Disabilities of Arm, Shoulder and Hand (Q-DASH) questionnaire. Grip and pinch strengths were measured using a hydraulic dynamometer and a hydraulic pinch gauge.

**Results:**

The AUSCAN pain, stiffness, function, total scores, and Q-DASH scores were significantly decreased in the splint group compared to the nonsplint group. Significant increments in grip and pinch strengths were detected in the splint group compared to the nonsplint group.

**Conclusion:**

The prefabricated carpometacarpal metacarpophalangeal immobilization splint is effective in improving pain, hand function, and hand strength in patients with thumb carpometacarpal osteoarthritis.

## 1. Introduction

Thumb carpometacarpal (CMC) joint osteoarthritis (OA) is the second most common hand OA. Patients with thumb CMC OA often present with pain, instability, and functional limitations. Pain and instability in the thumb may cause a reduction in the ability to perform activities of daily living such as grasping, pinching, and turning. The stability of the thumb CMC joint is essential to reduce pain and difficulty in daily living activities [1].

Treatment of thumb CMC OA consists of conservative therapeutic interventions and surgical interventions. Conservative therapy includes joint protection principles, splinting, pain control, exercise, nonsteroidal antiinflammatory drugs, corticosteroid injections, and physical therapy. Splinting is the mainstay of conservative therapy. The aims of splinting are to improve the stability of the thumb CMC joint by providing external support, to increase hand function, and to reduce pain. Increased stabilization of the first CMC joint prevents dorsal subluxation and further joint deformity, provides pain control, and maintains hand function [1–5].

The choice of splint design depends on which joint needs to be immobilized, the degree of OA, coexisting hand conditions, patient’s functional status, and patient preference [1,4]. Static splints are the most commonly used splints in thumb CMC OA [1]. The types of static splints are the wrist-CMC-MCP (metacarpophalangeal) immobilization splint (long opponens splint), the CMC-MCP immobilization splint (short opponens splint), and the CMC immobilization splint [2].

Evidence for the effectiveness of different types of splints in thumb CMC OA is limited [6–10]. The European League Against Rheumatism (EULAR) reported that placebo-controlled or nonsplint-controlled research evidence is required [11]. To our knowledge, there is only one randomized controlled trial with a nonsplint control in the literature. In this study, Rannou et al. studied the effects of a custom-made rigid CMC-MCP splint in patients with both early and advanced thumb CMC OA [10]. There is no study to assess the prefabricated CMC-MCP splint in the treatment of early-stage thumb CMC OA. The prefabricated CMC-MCP splint is readily available in varying sizes and more inexpensive than the custom-made CMC-MCP splint. Therefore, we aimed to evaluate the effects of a prefabricated CMC-MCP immobilization splint on pain, hand function, hand strength, and quality of life in patients with stage 1 or 2 thumb CMC OA.

In previous studies, stabilization of the thumb CMC joint in palmar abduction and the MCP joint in flexion with the short opponens splint was recommended in both early and advanced stage thumb CMC OA [12–14]. According to these studies, we preferred the prefabricated CMC-MCP immobilization splint in patients with early-stage OA. This type of splint provides adequate support to the CMC and MCP joints while allowing functional use of the hand. In addition, the prefabricated CMC-MCP splint is readily available in varying sizes and is less expensive than custom-made splints. To our knowledge, there is no study to assess the prefabricated CMC-MCP splint in the treatment of early-stage thumb CMC OA. Therefore, we aimed to evaluate the effects of the prefabricated CMC-MCP immobilization splint on pain, hand function, hand strength, and quality of life in patients with stages 1 or 2 thumb CMC OA.

## 2. Materials and methods

Sixty-six patients with 80 hands affected with stage 1 or 2 thumb CMC OA who were admitted to an outpatient hand clinic between July 2017 and January 2018 were enrolled in the study. The diagnosis of thumb CMC OA was made by a hand surgeon based on history, physical examination, and X-ray. Patients with thumb CMC OA were staged according to the Eaton-Littler-Burton classification system based on radiographic findings. The exclusion criteria were: 1) prior treatment for thumb CMC OA within the previous 6 months; 2) posttraumatic OA; 3) previous hand surgery; 4) inflammatory hand involvement; 5) neurologic hand involvement; 6) clinical signs of carpal tunnel syndrome, Dupuytren’s contracture, de Quervain tenosynovitis, and trigger finger; 7) peripheral vascular disease; 8) cognitive dysfunction; 9) skin disease interfering with wearing a splint; 10) pregnancy.

This study was performed with the approval of the local ethics committee in accordance with the ethical standards laid down in the 1964 Declaration of Helsinki and all subsequent revisions. All patients provided written informed consent to participate.

**Figure 1:**
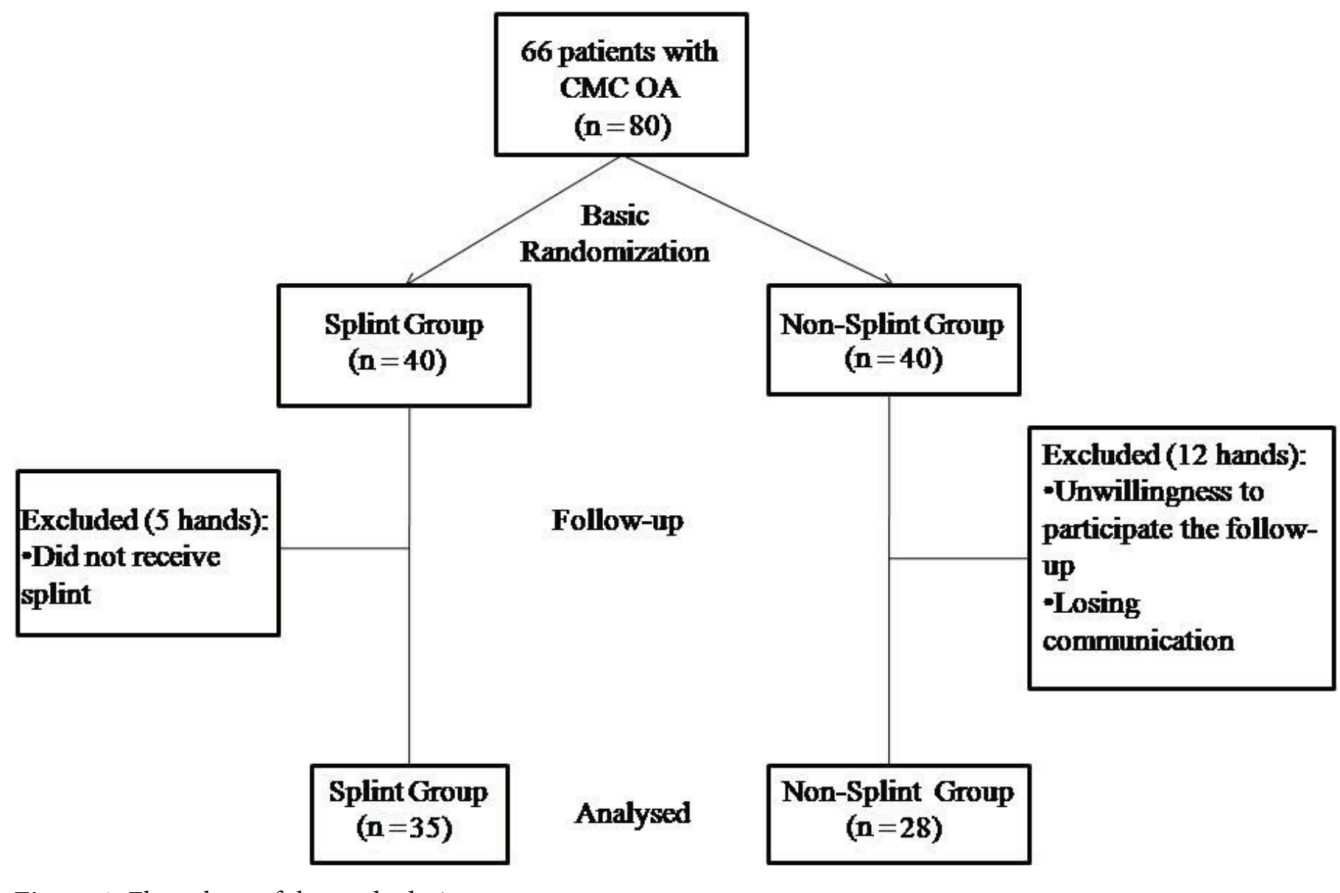
Flow chart of the study design.

Eighty hands were randomly assigned following a simple randomization procedure (computed random numbers) to a splint group (n = 40) and a nonsplint control group (n = 40). Seventeen hands were excluded from the study analysis. The remaining 63 hands were analysed. The flow chart of the study design is shown in Figure 1.

### 2.1. Interventions

**Figure 2:**
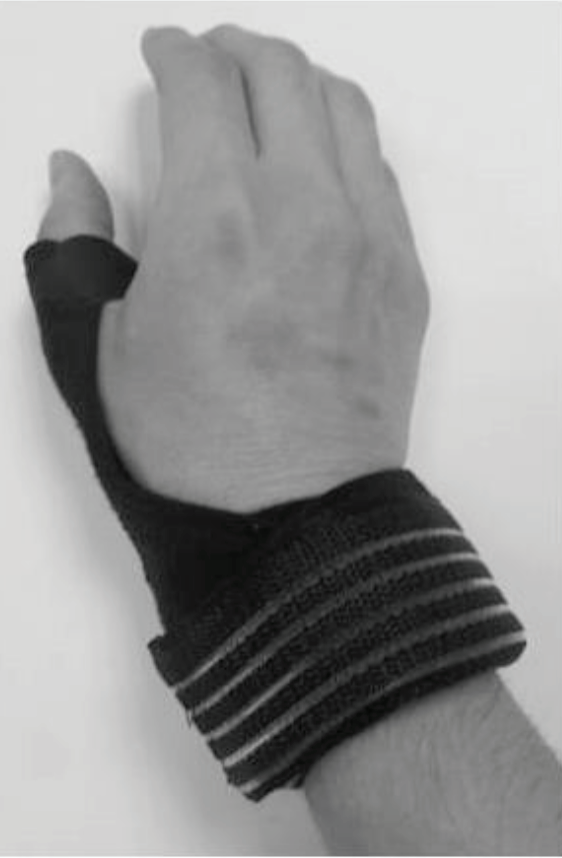
A prefabricated CMC-MCP immobilization splint.

The splint group was given a prefabricated splint program and oral information about how to accommodate activities of daily living (to use larger joints during daily living activities, to carry items on two flat hands rather than gripping with the fingers, to carry large or heavy items with two hands, to push items rather than carrying, to push up from a chair using the palm of the hand) throughout the study period. A prefabricated CMC-MCP immobilization splint (short opponens splint) was recommended for the patients in the splint group. This splint was made from neoprene with a removable metal stay. The metal stay extended along the thumb to support the first CMC joint in 30° palmar abduction and the MCP joint in 15° flexion (Figure 2). We assessed the suitability of the splint before starting usage. The patients were instructed to wear their splints all the time (daytime and night-time) as much as possible for the first 3 weeks and then only during painful activities for another 3 weeks. A maximum of 1500 mg of paracetamol per day was allowed to be taken for pain throughout the study.

The nonsplint control group received only oral information about how to accommodate activities of daily living throughout the study period. Paracetamol was allowed to be taken for pain throughout the study.

### 2.2. Assessments

The demographic and clinical characteristics of the patients at baseline were recorded. We used the Kapandji finger opposition test to assess the opposition of the thumb. The patients were instructed to touch the affected thumb to 10 points on the same hand.

Wrist/hand pain and functions were evaluated using the Australian/Canadian Osteoarthritis Hand Index (AUSCAN). It is a self-report assessment of pain (5 items), stiffness (1 item), and hand function (9 items) in OA of the hand. Each item is scored on a 5-point scale (0 = none, 4 = extreme) [15]. Higher scores indicate worse symptoms and function. The AUSCAN has been shown to be valid and reliable in measuring symptoms and hand function in hand OA [16].

We also used the Quick Disabilities of Arm, Shoulder and Hand (Q-DASH) questionnaire to assess hand functions. It has 11 items and each item is scored from 1 to 5. Higher scores indicate lower functional levels. The validity and reliability of the Turkish version of the Q-DASH have been done by Düger et al. [17].

Grip strength was measured using a Jamar hydraulic hand dynamometer. Lateral pinch, 2- point pinch, and 3-point pinch strengths were measured using a Jamar hydraulic pinch gauge (Bolingbrook, IL, USA). Patients were seated with shoulder adducted, elbow 90° flexed, forearm and wrist in neutral position. Patients were encouraged to press as firmly as possible. Three consecutive measurements were performed. The average of the three measurements was recorded in kilograms (kg) [18].

Patients’ quality of life was measured using the Nottingham Health Profile (NHP). It is a self-report questionnaire with 6 dimensions of health: physical mobility (8 items), pain (8 items), sleep (8 items), energy (8 items), social isolation (5 items), and emotional reaction (9 items). Patients were asked whether or not each item applied to them. Positive answers were given the appropriate weight according to their relative severity. Each dimension score ranges from 0 to 100. Lower scores indicate better quality of life. The Turkish validity and reliability study of the scale was performed by Kucukdeveci et al. [19].

All patients were asked to rate their overall satisfaction with the splint using a 10 cm visual analogue scale (VAS) at the end of the intervention.  

### 2.3. Follow-up

The authors called the patients weekly to assess adherence to wearing the splint. Patients were evaluated before the intervention and at the end of 6 weeks. All assessments were performed by the same clinician who was blind to the treatment.

### 2.4. Statistics

The sample size was determined based on a previous study. To achieve a 2 cm reduction on a 10 cm VAS pain scale in the splint group, we calculated at least 23 patients per group using the 5% significance level and 80% statistical power [20]. The Kolmogorov–Smirnov test was used to evaluate the distribution of normality. We performed the unpaired t-test for normally distributed data and the Mann–Whitney U test for nonnormally distributed data to compare the demographic characteristics, baseline measurements, and change scores between the groups. We used the paired samples t-test for normally distributed data and the Wilcoxon sign rank test for nonnormally distributed data to compare the difference between the baseline and posttreatment values within the groups. The Pearson Chi-square test was used to analyse categorical data. SPSS version 17 (SPSS Inc., Chicago, IL, USA) was used for all statistical analyses. P values less than 0.05 were considered to represent a significant difference.

## 3. Results

There was no significant difference in baseline demographic and clinical characteristics of the patients between groups. The baseline demographic and clinical characteristics of the patients are shown in Table 1. A total of 11% of patients in the splint group and 14% of patients in the nonsplint group used paracetamol for pain relief throughout the study. There was no significant difference between the groups (P = 0.04).

**Table 1:** Baseline demographic and clinical characteristics of the patients [(mean ± SD) or median (min-max)].

	Splint group (n = 35)	Nonsplint group (n = 28)	P
Age	56.1 ± 7.5	56.6 ± 9.2	0.67
Sex Male Female	8.3% 91.7%	10.7% 89.3%	0.55
Education (years)	6.5 ± 1.2	6.8 ± 1.4	0.72
Occupation Housewife Worker Retired	77.8% 13.9% 8.3%	78.6% 14.3% 7.1%	0.98
Dominant hand side Right Left	97.2% 2.8%	92.9% 7.1%	0.58
Symptomatic hand Dominant Non-dominant	50% 50%	46.4% 53.6%	0.53
Symptom duration (months)	12(3-60)	12(1-36)	0.70
AUSCAN Pain Stiffness Function Total	13.5 ± 3.7 1(0–4) 24.1 ± 7.8 39.2 ± 10.8	13.7 ± 4.1 1(0–3)23.1 ± 7.6 37.9 ± 11.4	0.88 0.81 0.91 0.95
Q-DASH	53.2 ± 16.1	48.2 ± 18.3	0.28
Kapandji (0–10)	9.5 ± 1.6	9.8 ± 0.4	0.07
Grip strength (kg)	14.3 ± 6.7	13.8 ± 5.7	0.41
Lateral pinch (kg)	6.4 ± 1.9	6.2 ± 1.9	0.99
2-point pinch (kg)	6.1 ± 1.9	5.6 ± 1.8	0.61
3-point pinch (kg)	6.2 ± 2.1	5.7 ± 1.8	0.32
NHP Pain Energy level Emotional reaction Sleep Social isolation Physical mobility Total	80(0–100) 100(0–100) 83.7(0–100) 64.4 ± 33.1 42(0–100) 45.1 ± 29.9 375.9 ± 148.9	80(0–100) 100(0–100) 46(0–100) 41.4 ± 35.1 17.5(0–100) 43.9 ± 25.1 309.1 ± 151.1	0.59 0.490.06 0.45 0.29 0.47 0.87

SD: standard deviation; AUSCAN: Australian Canadian Osteoarthritis Hand Index, Q-DASH: Disabilities of Arm, Shoulder and Hand questionnaire, NHP: Nottingham Health Profile.

The AUSCAN pain, stiffness, function, and total scores were significantly decreased in the splint group at the end of the treatment (P < 0.05). In the nonsplint group, there were no statistically significant differences in the AUSCAN pain, stiffness, function, and total scores at the end of treatment. The Q-DASH scores were also decreased in the splint group after treatment (P < 0.05), whereas no significant changes occurred in the nonsplint group. We did not find a significant change in Kapandji scores of patients after treatment in both groups. The AUSCAN, Q-DASH, and Kapandji scores of the groups are shown in Table 2.

**Table 2:** The AUSCAN, Q-DASH, Kapandji, NHP, hand grip strength and pinch strength values of the patients[(mean ± SD) or median (min-max)]

	Splint Group (n=35)			Non-Splint Group (n=28)		
	Pre-treatment	Post-treatment	p	Pre-treatment	Post-treatment	p
AUSCAN Pain	13.5±3.7	7.3±4.1	<0.001*	13.7±4.1	12.3±5.3	0.06
AUSCAN Stiffness	1(0-4)	0(0-4)	<0.001*	1(0-3)	0(0-3)	0.81
AUSCAN Function	24.1±7.8	11.8±7.2	<0.001*	23.1±7.6	20.4±9.4	0.11
AUSCAN Total	39.2±10.8	19.3±11.2	<0.001*	37.9±11.4	33.6±15.2	0.06
Q-DASH	53.2±16.1	25.2±15.8	<0.001*	48.2±18.3	44.6±22.6	0.23
Kapandji (0-10)	9.5±1.6	9.5±1.2	0.07	9.8±0.4	9.8±0.4	
Grip strength (kg)	14.3±6.7	17.2±6.6	<0.001*	13.8±5.7	14.1±5.8	0.47
Lateral pinch (kg)	6.4±1.9	7.8±2.7	0.002*	6.2±1.9	6.1±2.2	0.33
2-point pinch (kg)	6.1±1.9	7.4±2.9	0.001*	5.6±1.8	5.5±1.6	0.55
3-point pinch (kg)	6.2±2.1	7.6±3.2	0.002*	5.7±1.8	5.8±2.2	0.42
NHP Pain	80(0-100)	53.4(0-100)	0.004*	80(0-100)	59.5(0-100)	0.13
NHP Energy level	100(0-100)	63.2(0-100)	0.01*	100(0-100)	100(0-100)	0.06
NHP Emotion reaction	83.7(0-100)	38.7(0-100)	<0.001*	46(0-100)	42.4(0-100)	0.07
NHP Sleep	64.4±33.1	39.9±34.1	<0.001*	41.4±35.1	39.3±35.1	0.59
NHP Social isolation	42(0-100)	0(0-100)	0.002*	17.5(0-100)	0(0-100)	0.13
NHP Physical mobility	45.1±29.9	36.1±20.6	0.02*	43.9±25.1	40.8±21.8	0.22
NHP Total	375.9±148.9	257.8±142.1	<0.001*	309.1±151.1	273.1±156.4	0.009*

AUSCAN: Australian Canadian Osteoarthritis Hand Index, Q-DASH: Disabilities of Arm, Shoulder and Hand questionnaire, NHP: Nottingham Health Profile, SD: standard deviation, *: statistically significant

In the splint group, we detected significant increments in grip strength, lateral pinch, 2-point pinch, and 3-point pinch strengths at the end of the treatment (P < 0.05). However, no significant increments in grip strength and pinch strength were detected in the nonsplint group after treatment. Grip strength and pinch strength of the groups are shown in Table 2.

The NHP pain, energy level, emotional reaction, sleep, social isolation, physical mobility, and total scores of the splint group decreased significantly after the treatment (P < 0.05). Only the NHP total score decreased significantly in the nonsplint group after the treatment (P < 0.05). The change in NHP total score for the splint group was significantly better than that for the nonsplint group (–117.7 ± 59.5 and –35.9 ± 7.1, respectively; P = 0.002). The NHP scores of the groups are shown in Table 2.

The mean satisfaction score of patients with

splint usage was 7.3 ± 1.7 (0–10 cm VAS).

Scores greater than or equal to 5 were accepted as high satisfaction with the splint. According to this, 77% of the patients in the splint group had a high satisfaction level. A total of 23% of the patients in the splint group reported that the splint was uncomfortable and restricted their daily activities.

## 4. Discussion

Although different types of splints were evaluated in the previous studies, what is the optimal splint type in the treatment of thumb CMC OA is controversial [1,3]. To the best of our knowledge, this is the first study to compare the effects of a prefabricated CMC-MCP immobilization splint for a duration of 6 weeks with no splinting in patients with early-stage thumb CMC OA. In the present study, we detected that the prefabricated CMC-MCP immobilization splint is an effective treatment intervention for improving pain, hand function, and quality of life in patients with early-stage thumb CMC OA.

There is no evidence on which type of splint was more effective in pain relief and enhancing the function of the thumb CMC OA. Bongi et al. splinted 13 patients with thumb CMC OA in a custom-made short opponens splint for 1 month and detected significant pain relief at the end of the intervention [9]. Weiss at al. evaluated the effects of rigid custom-made CMC immobilization splint and prefabricated CMC-MCP immobilization splint in 25 hands with stage 1 or 2 thumb CMC OA. Both pain and function were improved more with the prefabricated CMC-MCP immobilization splint [7]. These findings are consistent with the results of the present study, in which the prefabricated CMC-MCP immobilization splint provided significant pain relief and functional improvement compared with no splint. On the contrary, Sillem et al. showed that custom-made CMC immobilization splint was significantly better than prefabricated CMC-MCP immobilization splint to reduce pain. They showed no improvement in hand function after wearing the splints for 4 weeks [3]. Rannou et al. found no significant differences in pain and disability between the custom-made rigid CMC-MCP immobilization splint group and the nonsplint group at 4 weeks [10]. The different results were attributed to the differences in sample size, splint materials, splint wearing schedule, stage of thumb CMC OA, and measured variables.

The evidence regarding the use of splinting to improve hand strength is insufficient [3,7,10]. We found significant increments in grip strength, lateral pinch strength, 2-point pinch strength, and 3-point pinch strength in the splint group compared to the nonsplint group at the end of the sixth week. We believe the increase in grip and pinch strengths observed in the splint group was due to a reduction in pain and an improvement in thumb CMC joint stabilization. Our results differ from the previous studies in regard to the effect of splinting on the grip and pinch strengths. Weiss et al. found that both custom-made CMC immobilization and prefabricated CMC-MCP immobilization splints did not significantly alter the pinch strengths as compared with pretreatment values [7]. Sillem et al. reported no improvement in grip and pinch strengths after wearing the prefabricated CMC-MCP immobilization splint in patients with thumb CMC OA [3]. Rannou et al. did not detect a significant difference in pinch strength between the custom-made rigid CMC-MCP immobilization splint group and the nonsplint group [10]. It was difficult to compare the results of previous studies with the present study because of differences in study samples, splint materials, and duration of splint use.

Besides pain, physical function, and strength, it is also important to assess the quality of life of the patients with hand and thumb OA. Hand and thumb OA has a high impact on the quality of life of patients [21]. To our knowledge, there is no study in the literature to evaluate the effects of splinting on the quality of life of patients with thumb CMC OA. We assessed the effects of splinting on the quality of life of patients using NHP and detected that splinting is effective in improving the quality of life of patients due to pain relief and increased hand function.

In the present study, the patients rated their splint satisfaction using a 10-cm VAS. The mean satisfaction score of the patients was 7.3. When a score of 5.0 or more was accepted as high satisfaction, 77% of patients had high satisfaction with the prefabricated CMC-MCP immobilization splint. Seventy-seven percent of patients reported that daily activities were easier to perform while wearing the splint, whereas 23% of patients reported difficulty in performing daily activities. This result is in line with those reported by other studies [3,7]. Weiss et al. reported that three-quarters of patients with thumb CMC OA preferred the prefabricated CMC-MCP immobilization splint [7]. In another study, 63% of patients preferred the prefabricated CMC-MCP immobilization splint to the custom-made CMC immobilization splint due to its comfortable usage [3].

The information about wearing splint schedules for thumb CMC OA in the literature is contradictory [8,9,13]. Swigart et al. offered to wear the splint continuously for 3 weeks and then only during the heaviest activities for 3 weeks [8]. Bongi et al. instructed to wear the splint for 5–6 h per day and at night during 4 weeks, then during heavy activities only [9]. Glickel recommended the splint only during the day for pain relief [13]. In the present study, patients were instructed to wear the splint all the time as much as possible during the first 3 weeks for immediate reduction of pain. After this period, the splint was recommended only during activities of daily living that cause pain to support the hand functions and to reduce pain. We found that splinting is a well-tolerated conservative treatment in patients with thumb CMC OA.

The study has some limitations. First, the follow-up time of the study (6 weeks) was short. Longer-term follow-up of patients after the end of the scheduled treatment period will provide more important information on the persistent effects. Second, we did not evaluate the effects of another type of splint in patients with thumb CMC OA. A comparison of the effects of different types of splints with no splinting will provide a more valuable contribution to the literature.

## 5. Conclusions

In conclusion, we evaluated the effects of a prefabricated CMC-MCP immobilization splint in patients with thumb CMC OA. We detected that this type of splint is effective in improving pain, hand function, grip strength, pinch strength, and quality of life at the end of a 6-week usage period. Future randomized controlled studies with larger sample sizes and longer follow-up periods are needed to confirm the effects of splinting in the treatment of thumb CMC OA.

## Informed Consent

The study protocol received institutional review board approval. All participants provided informed consent in the format required by the relevant authorities.
